# Late Presentation of Giant Intrathoracic Neurofibroma with Significant Mediastinal Shift: A Case Report and Review of the Literature

**DOI:** 10.1155/2013/619729

**Published:** 2013-03-20

**Authors:** Emeka B. Kesieme, Andrew E. Dongo, Christopher Affusim, Georgi Prisadov, Kelechi Okonta, Clement Imoloamen

**Affiliations:** ^1^Department of Surgery, Irrua Specialist Teaching Hospital, PMB 8, Irrua, Edo State, Nigeria; ^2^Department of Family Medicine, Irrua Specialist Teaching Hospital, PMB 8, Irrua, Edo State, Nigeria; ^3^Department of Thoracic Surgery, Hospital Bremen-Ost, Osterholzer Landstrasse 51, Bremen, Germany; ^4^Department of Surgery, University of Port Harcourt Teaching Hospital, PMB 6173, Port Harcourt, River State, Nigeria

## Abstract

Intrathoracic tumours in patients with Von Recklinghausen's disease have been widely reported, but there are very few cases of reported intrathoracic giant benign neurofibroma with marked mediastinal shift and superior vena cava syndrome. Patients that present with this pathology should be adequately investigated. Surgical resection has been considered curative.

## 1. Introduction

Patients with neurofibromatosis type I (Von Recklinghausen's disease) are prone to developing benign and malignant tumours of the thorax in addition to its cutaneous, orthopedic, and neurologic manifestations.

Benign intrathoracic nerve sheath tumours include neurofibroma, schwannomas, and ganglioneuroma arising either de novo or from transformation of neurofibroma [1].

Neurofibromas involving the vagus nerve [2, 3], recurrent laryngeal nerve [4], and phrenic nerve [5] have all been reported. A rapidly growing benign intrathoracic tumour arising from the sympathetic trunk after lobectomy has been reported [6]. However, only very few cases of giant intrathoracic neurofibroma with mediastinal shift or superior vena cava syndrome have been reported [7–9].

## 2. Case Report

A 23-year-old student presented to us with left side chest pain of 16 months duration and bulging left anterior chest wall of 6 months duration.

**Figure 1:**
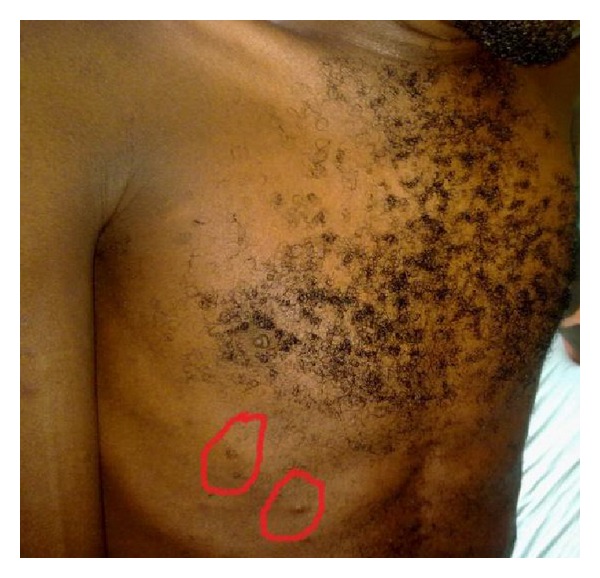
Multiple cutaneous neurofibroma and café-au-lait spots (circled in red).

General examination revealed multiple cutaneous neurofibroma and café-au-lait spots (Figure 1). Chest examination revealed a bulging left anterior chest wall and thoracic kyphoscoliosis. Trachea was deviated to the right, and apex beat was located at the 5th right intercostal space, midclavicular line. The entire anterior chest wall was dull to percussion, and breath sounds were absent. Neck veins were distended with facial and left upper limb oedema.

**Figure 2:**
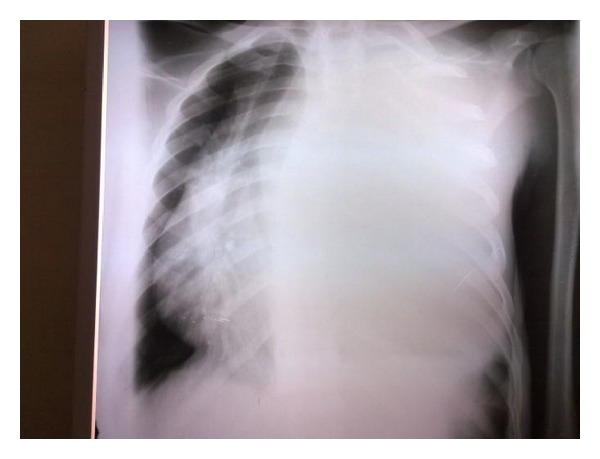
Chest radiograph showing homogenous opacification of the entire left hemithorax with marked mediastinal shift to the right.

Chest radiograph revealed homogenous opacification of the entire left hemithorax with marked mediastinal shift to the right (Figure 2).

**Figure 3:**
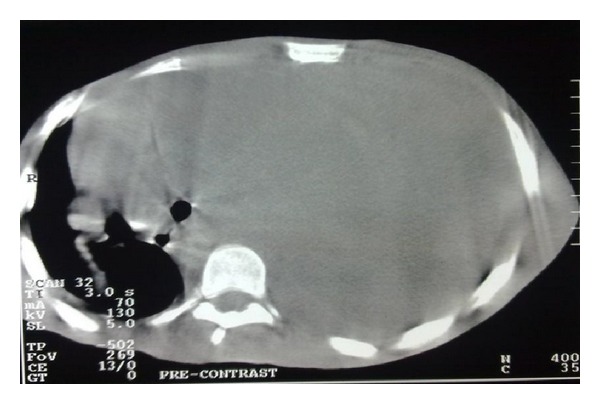
Chest CT scan showing non-enhancing mass, isodense to surrounding muscle completely occupying the entire left hemithorax.

Chest CT scan revealed nonenhancing mass, isodense to surrounding muscle completely occupying the entire left hemithorax (Figure 3). Incisional biopsy was taken via left minithoracotomy. Macroscopy revealed whitish tissue that was soft in consistency. Microscopy revealed an unencapsulated benign neoplastic lesion composed of nodules of interwoven fibromyxoid connective tissue stroma. Features were in keeping with neurofibroma with no evidence of malignancy. 

Patient's condition deteriorated, and he died from respiratory insufficiency as he was being worked up for thoracotomy.

## 3. Discussion 

Neurofibromatosis type I (NF-1) (Von Recklinghausen's disease) is an autosomal dominant disease with complete penetrance and extremely variable expression, with an incidence of approximately 1 in 4000 life birth.

The NF-1 gene has been mapped to chromosome 17p11.2 and cloned [10, 11]. The gene product neurofibroma functions as tumour suppressor genes. Loss of neurofibromin leads to an increased risk of developing benign tumours in affected individuals.

Our patients showed classical features of NF-1, which include more than 6 café-au-lait spots present at birth and multiple neurofibromas. Other features that make up the diagnostic criteria include first degree family relative of NF-1, Lisch nodule of iris, axillary or groin freckling, optic pathway glioma, bony dysplasia of the sphenoid bone, and pseudoarthrosis. Thoracic kyphoscoliosis may have been due to the effect of the tumour since it developed with symptoms of the tumour.

Neurofibromas arise from Schwann cells and fibroblast and may arise in any peripheral nerves; hence, thoracic manifestation of Von Recklinghausen's disease may involve the ribs, chest wall, lungs, and mediastinum.

Intrathoracic neurogenic tumours are predominantly neurofibromas, benign schwannomas, or sarcoma, mostly developing on the course of intercostal nerves or from ganglia or nerves of sympathetic truck. However, there are also reports in the literature of neurofibromas arising from trachea and oesophagus [12, 13].

Malignant peripheral nerve sheath tumours can arise de novo but have also been reported following malignant transformation of mediastinal neurofibromas [1]. Hence, if a giant intrathoracic neurogenic tumour is identified, its malignant potential should be evaluated based on hypercellularity, the presence of necrosis, its cytological atypia, mitotic index, the proliferation index (k-67 expression), and p53 positivity in immune histochemical stains [8].

Presenting features may depend on the location, size, and histology. They may be asymptomatic but giant tumours as presented earlier will present with features in keeping with compression of nearby structures.

Hourglass-shaped tumours of the nerve roots often present with medullary compression signs, while those of the pulmonary apex present with an anterior mediastinal compression [12]. Superior vena cava syndrome in the index patient presented as facial, neck, and upper limb edema and distended neck veins.

Tumors of the phrenic nerve may provoke dyspnea by paralysis of the corresponding hemidiaphragm; those of the vagus nerve are associated with dysphagia, bradycardia, or diarrhea [12]. Hoarseness in the presence of suspected intrathoracic neurogenic tumour is not pathognomonic of malignancy [14]. Paralysis of the vocal cords can result from pressure on the recurrent laryngeal nerve.

Chest radiograph may reveal homogenous or heterogenous mass, mediastinal and bony deformities.

Computerized tomography scan and magnetic resonance imaging techniques have greatly enhanced the accuracy of diagnosis of intrathoracic tumours. The latter allows precise identification of spinal cord involvement, intraspinal extension, and relation to other critical structures [15].

The coexistence of rare retroperitoneal neurogenic tumours should be recognized during the evaluation and management of NF-1 patients with suspected mediastinal neurogenic tumours [15].

Malignant peripheral nerve sheath tumours and intrathoracic meningocele are possible differentials in cases of giant intrathoracic tumours in patients with cutaneous neurofibromatosis type 1 [16, 17].

Surgery is the mainstay of treatment, and resection should be complete and in one stage.
